# Phenotypic and molecular reanalysis of a cohort of patients with monogenic diabetes reveals a case of partial lipodystrophy due to the A8344G mutation in the mitochondrial DNA

**DOI:** 10.20945/2359-4292-2023-0084

**Published:** 2024-06-19

**Authors:** Pedro Campos Franco, Michelle Patrocinio, Aline Dantas Costa-Riquetto, Augusto Cezar Santomauro, Larissa Garcia Gomes, Milena G. Teles

**Affiliations:** 1 Hospital das Clínicas Faculdade de Medicina Universidade de São Paulo São Paulo SP Brasil Grupo de Diabetes Monogênico, Unidade de Endocrinologia Genética (LIM25), Unidade de Diabetes, Hospital das Clínicas, Faculdade de Medicina, Universidade de São Paulo, São Paulo, SP, Brasil; 2 Hospital Santa Marcelina São Paulo SP Brasil Hospital Santa Marcelina, São Paulo, SP, Brasil; 3 Hospital das Clínicas Faculdade de Medicina Universidade de São Paulo São Paulo SP Brasil Unidade de Desenvolvimento, Hospital das Clínicas, Faculdade de Medicina, Universidade de São Paulo, São Paulo, SP, Brasil

## Abstract

Familial partial lipodystrophy (FPLD) is a very rare genetic disease characterized by insulin resistance due to a loss of subcutaneous fat from the extremities together with a progressive storage of fat around the face and neck and inside the abdomen. In over 50% of cases, molecular genetic testing reveals pathogenic variants in two nuclear genes, LMNA and PPARG. The case reported here refers to a woman phenotypically diagnosed with FPLD, who presented with diabetes and multiple cervical lipomatosis and in whom no variant had been found in the nuclear genes classically associated with this syndrome that could explain her phenotype. Genetic sequencing using a target panel containing 48 nuclear genes related to monogenic diabetes plus the whole mitochondrial genome revealed the mitochondrial variant m.A8344G in 84.1% heteroplasmy. Following molecular diagnosis, her phenotype was expanded with the recognition of additional clinical characteristics: mild sensorineural hearing loss, proximal myopathy, fatigue, cognitive impairment, sensory ataxia, cardiac abnormalities and, finally, muscle biopsy findings compatible with mitochondrial disease. Therefore, careful and detailed phenotypic and genotypic reanalysis proved crucial in improving molecular diagnosis in FPLD.

## INTRODUCTION

Lipodystrophies are characterized by a selective lack of adipose tissue. Familial partial lipodystrophy (FPLD) involves insulin resistance due to the loss of subcutaneous fat from the extremities and lower trunk and its progressive accumulation in the face and around the neck (1). Heterozygous mutations in the *LMNA* and *PPARG* nuclear genes have been reported to account for approximately 50% of cases in which the genetic cause is identified (2). The mitochondrial A8344G variant is classically described as being the cause of the Myoclonic Epilepsy with Ragged-Red Fibers (MERRF) syndrome, a multi-systemic mitochondrial disease first described in 1990 (3) and characterized by myoclonus, seizures, cerebellar ataxia and mitochondrial myopathy. Since then, an expansion of the phenotypic spectrum has been reported (4), and this variant has been associated with other phenotypes including lipomatosis, diabetes, deafness and psychiatric disorders. The objective of this case report was to describe a mitochondrial variation in a patient with a clinical diagnosis of FPLD.

## CLINICAL PRESENTATION

**Figure 1:**
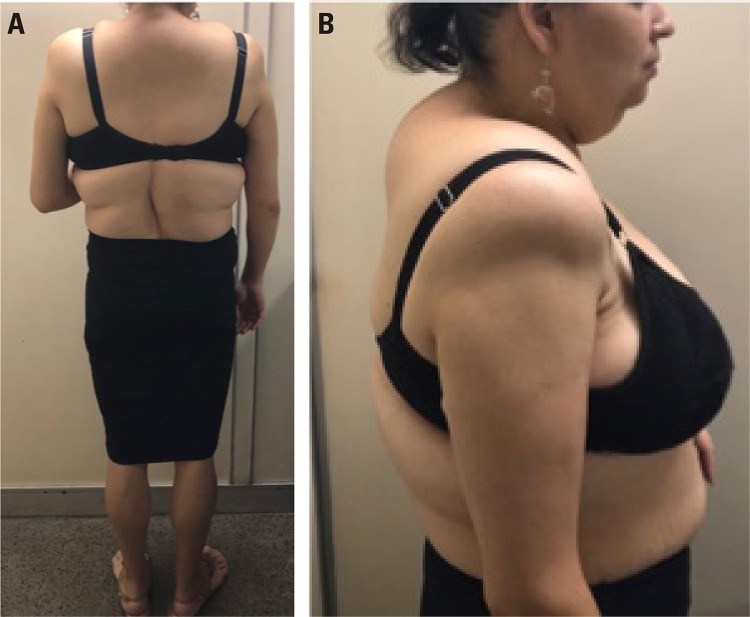
(A) Predominantly truncal fat distribution, with little subcutaneous tissue in the lower limbs. (B) Presence of fat pad in the cervical region and marked absence of fat in the right upper limb.

**Figure 2:**
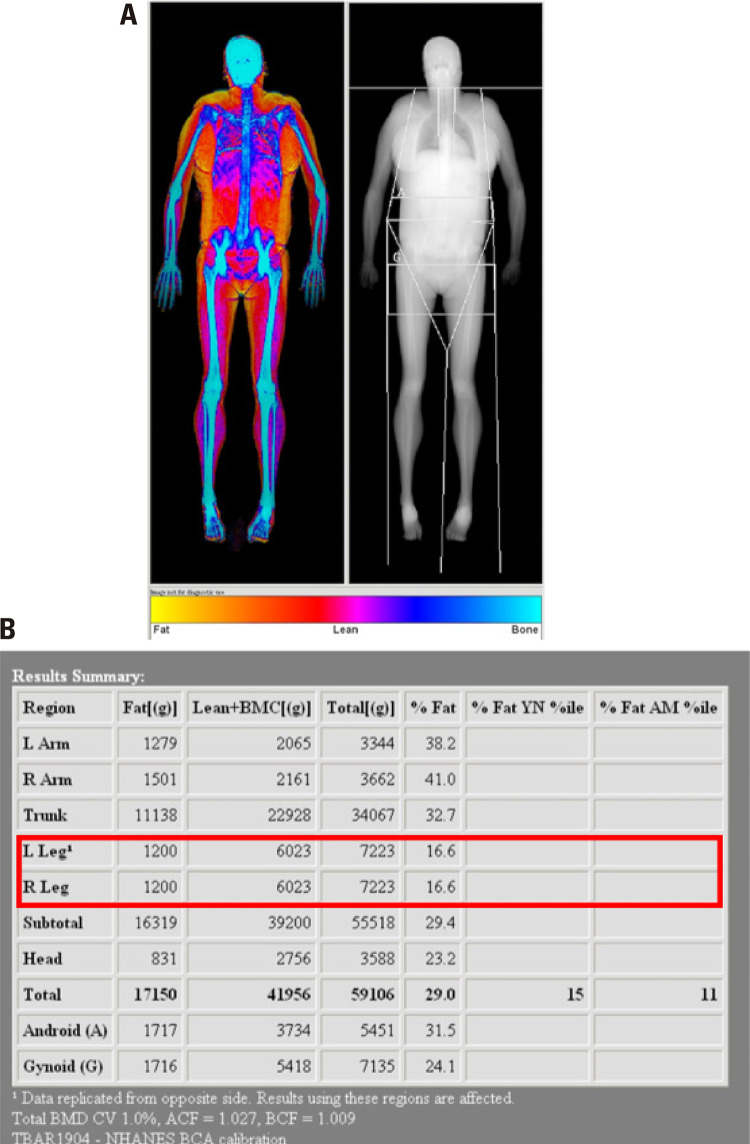
(A) Densitometry image generated by specific software (Fat Shadow) used to visualize body fat. Note the loss of fat in the extremities and the accumulation of fat in the truncal region. Color spectrum represents tissue density (yellow = fat; red/purple = muscle; blue = bone). (B) Differential quantification of fat by body region obtained by densitometry. Red square pinpoints the low concentration of fat in the lower limbs characteristic of FPLD (5).

The patient described here is a 38-year-old woman phenotypically defined as having FPLD. Between 10 and 15 years of age, a change in her body fat distribution became noticeable, with prominent fat storage in the supraclavicular fossa and dorsocervical fat pad as shown in Figures 1A, 1B and 2A. The low concentration of fat in the lower limbs that is a general characteristic of FPLD is illustrated in Figure 2B (5). At 18 years of age, there were signs of insulin resistance, facial hirsutism, irregular menstrual cycles and androgenetic alopecia. In addition, ultrasonography revealed enlarged, micro-polycystic ovaries, leading to a diagnosis of polycystic ovary syndrome. The patient was diagnosed with diabetes mellitus (DM) at 28 years of age. Her body weight was normal. She was initially treated with oral anti-diabetic drugs for two years, at which time insulin had to be added to the treatment regimen. Concomitantly, papillary thyroid carcinoma, hypertension, dyslipidemia and depression were diagnosed. Metabolic abnormalities are now being treated with a combination of metformin, pioglitazone, fenofibrate, statins and high doses of insulin (1.2 IU/kg/day). With regards to glucose homeostasis, endogenous insulin secretion remained adequate as shown by a C-peptide measurement of 1.94 ng/mL and HbA1c of 6.1, glucose of 112 mg/dL and creatinine clearance of 71.4 mL/min/1.73 m^2^ according to the Chronic Kidney Disease Epidemiology Collaboration (CKD-EPI) equation. The lipid profile showed levels of total cholesterol of 216 mg/dL, triglycerides 245 mg/dL, HDL 51 mg/dL and LDL 131 mg/dL. Finally, there is a significant history of DM on the maternal side of the patient’s family. Her 65-year old mother was diagnosed with DM during pregnancy at 35 years of age and is in treatment with oral anti-diabetic drugs. The patient has two maternal uncles who have been diagnosed with DM and are being treated with insulin therapy, and a maternal aunt also diagnosed with DM and in treatment with anti-diabetic drugs. She has two brothers of 30 and 47 years of age, respectively, neither of whom has DM.

### Genetic analysis

The institutional ethics committee approved the study protocol, assigning it the reference number 70637. Prior to her participation, written informed consent was obtained from the patient.

In view of the DM phenotype associated with insulin resistance, together with the abnormal fat distribution characteristic of FPLD (1), molecular analysis was performed using a target panel containing 48 genes (6) related to monogenic diabetes, including those linked to FPLD (*LMNA* and *PPARG, PLN1* and *AKT2*), plus the entire mitochondrial genome. DNA extracted from the patient’s blood had initially been sequenced and extensively analyzed in 2016, with no causative variant being found in the classical nuclear genes associated with FPLD. However, because the patient had the typical phenotype of the syndrome, and based on the formal recommendation of the American College of Medical Genetics and Genomics (ACMG) (7) to perform biennial genetic reanalysis, in 2021 a decision was made to systematically reanalyze data in our records and search for any overlooked genetic modifications. By investigating beyond the classical nuclear genes associated with FPLD and revisiting information in the literature on mitochondrial variants related to DM, the mitochondrial variant m.A8344G was found in 84.1% heteroplasmy.

## DISCUSSION

Since 2019, the ACMG has recommended variant-level reevaluation and case-level reanalysis every two years as an effective means of maximizing clinical impact while minimizing the burden to the laboratory and healthcare system (7). From a genotype point of view, accelerated advancements in molecular diagnostics and their progressively lower cost have led to rapidly increasing and constantly evolving knowledge in the field of genetics. Therefore, classifying variants has become a less static process. Phenotypic presentation also does not remain static, since the individual’s clinical presentation changes over time, with new characteristics emerging that may prove crucial to defining the case. Furthermore, the phenotypic spectrum of a disease can expand as other individuals with specific genotypes are identified.

In the present case, reanalysis not only allowed a more detailed molecular diagnosis to be reached, but also permitted a better characterization of the patient’s phenotype. Once m.A8344G was detected, this information was applied at patient level, with multiple medical specialties then participating in the investigative and care process, characterizing good practice towards precision medicine. Consequently, the patient was further diagnosed with mild sensorineural hearing loss, multiple cervical lipomatosis, proximal myopathy, fatigue, cognitive impairment, sensory ataxia, and cardiac abnormalities (isolated atrial and ventricular extrasystoles). Ultimately, muscle biopsy findings were compatible with mitochondrial disease.

Since m.A8344G was originally described as a cause of syndromic disease affecting the central nervous system (3), other descriptions of such cases have been published in the literature. A broader review (4) describes common neurological manifestations (around 88% of the patients evaluated had an abnormal electroencephalogram, 70% cerebellar ataxia and 61% seizures) to the detriment of the less common metabolic involvement (around 10% had DM). The present case is different, not only since the metabolic phenotype involved suggested a need for molecular reanalysis, but also because of the absence of neurological manifestations (normal electroencephalogram, no seizures, no cerebellar ataxia) up to that point.

The mechanisms through which mitochondrial dysfunction can generate insulin resistance were thoroughly reviewed in a recent study (8). The most likely hypothesis is that the decreased effectiveness of the oxidative phosphorylation pathway results in poorer oxidative capacity that hampers beta oxidation in muscle tissue, resulting in muscle insulin resistance; therefore, the adipose tissue would not be involved. Nevertheless, as shown by Kobayashi and cols. (9), adequate mitochondrial function is pivotal for maintaining healthy adipose tissue. Those investigators reported that defects in the mitochondrial unfolded protein response (UPR^mt^) in adipocytes can induce white adipose tissue atrophy and resistance to diet-induced obesity. From a clinical point of view, a recent review (10) highlighted the important role of nuclear genes (*MFN2* and *LIPE*) in the adequate mitochondrial function. Pathogenic variants in both of these genes are a known cause of partial lipodystrophy associated with pseudo-lipomatous regions. However, although that hypothesis is plausible, the same paper also stated that neither metabolic phenotype nor potential partial lipodystrophy signs were systematically investigated in patients harboring mtDNA mutations, particularly in those harboring the m.8344A>G variant.

With respect to pancreatic beta cell function, the persistence of robust insulin secretion, confirmed by C-peptide measurement, shows that insulin resistance played a core role in the physiopathology of this DM and not insulinopenia, previously believed to be the principal mechanism in the development of hyperglycemia in individuals with mitochondrial DM (11). This finding is in agreement with the current literature on the subject (12), which shows a broad phenotypic spectrum of DM associated with mitochondrial abnormalities, ranging from mild cases controlled with anti-diabetic drugs to cases in which insulinopenia is more severe, requiring insulin therapy.

The FPLD phenotype is classically described as having an autosomal dominant inheritance pattern; therefore, there tends to be a high incidence of similar cases in the family. Notwithstanding, in the case reported here, the history of family members with a similar fat distribution pattern was unavailable. Therefore, within these limitations, it may be concluded that this case would appear to resemble the FPLD phenotype because of its predominant feature of reduced subcutaneous fat in the lower limbs, as documented at dual-energy x-ray absorptiometry (DXA).

This case shows that direct defects in the mitochondrial genome can be a cause of severe insulin-resistant phenotypes such as partial lipodystrophy. Therefore, m.A8344G should be tested not only in familial multiple symmetric lipomatosis, but also in individuals with FPLD. Further studies and case reports are necessary to determine whether this condition is a phenocopy or whether a new molecular cause of disease has indeed been identified.
